# Retraction Note: Helicobacter pylori strains isolated from raw poultry meat: frequency and molecular characteristics

**DOI:** 10.1038/s41598-024-60110-w

**Published:** 2024-04-23

**Authors:** Tohid Piri-Gharaghie, Ghazal Ghajari, Shakiba Tolou-Shikhzadeh-Yazdi, Mona Aghassizadeh-Sherbaf, Sahar Khorsand-Dehkordi

**Affiliations:** 1 Biotechnology Research Center, Faculty of Basic Sciences, Islamic Azad University, East-Tehran Branch, Tehran, Iran; 2 Department of Cell and Molecular Biology, Faculty of Biological Sciences, Kharazmi University, Tehran, Iran; 3 Department of Biology, Faculty of Sciences, Islamic Azad University, Mashhad Branch, Mashhad, Iran; 4 Department of Biology, Faculty of Basic Sciences, Islamic Azad University, East-Tehran Branch, Tehran, Iran; 5 Department of Biology, Faculty of Basic Sciences, Islamic Azad University, Shahrekord Branch, Shahrekord, Iran

Retraction of: *Scientific Reports* 10.1038/s41598-023-38374-5, published online 10 July 2023

Editors have retracted this Article.

After publication of this Article, concerns were raised about overlap in text and data with a prior publication with no common authors^1^. The Authors provided the raw data for the paper, but the subsequent analysis of this information identified discrepancies between the gel images provided and the data reported in the Article. The Editors therefore no longer have confidence in the reliability of the Article's findings or conclusions.

Tohid Piri‑Gharaghie, Shakiba Tolou‑Shikhzadeh‑Yazdi, Mona Aghassizadeh‑Sherbaf, and Sahar Khorsand‑Dehkordi disagree with this retraction. Ghazal Ghajari did not respond to correspondence from the Editors about this retraction.
